# Inter-modality assessment of medial temporal lobe atrophy in a non-demented population: application of a visual rating scale template across radiologists with varying clinical experience

**DOI:** 10.1007/s00330-021-08177-1

**Published:** 2021-07-30

**Authors:** Claes Håkansson, Ashkan Tamaddon, Henrik Andersson, Gustav Torisson, Gustav Mårtensson, My Truong, Mårten Annertz, Elisabet Londos, Isabella M. Björkman-Burtscher, Oskar Hansson, Danielle van Westen

**Affiliations:** 1 Department of Imaging and Function, Skåne University Hospital, Lund, Sweden; 2 Department of Clinical Sciences, Diagnostic Radiology, Lund University, Lund, Sweden; 3 Department of Translational Medicine, Clinical Infection Medicine, Lund University, Malmö, Sweden; 4 Department of Clinical Sciences Malmö, Clinical Memory Research Unit, Lund University, Malmö, Sweden; 5 Department of Neurobiology, Care Sciences and Society, Karolinska Institute, Stockholm, Sweden; 6 Memory Clinic, Skåne University Hospital, Malmö, Sweden; 7 Department of Radiology, Clinical Sciences, Sahlgrenska Academy, University of Gothenburg, Gothenburg, Sweden

**Keywords:** Cognitive dysfunction, Magnetic resonance imaging, Tomography, X-ray computed, Radiologists, Consensus

## Abstract

**Objectives:**

To assess inter-modality agreement and accuracy for medial temporal lobe atrophy (MTA) ratings across radiologists with varying clinical experience in a non-demented population.

**Methods:**

Four raters (two junior radiologists and two senior neuroradiologists) rated MTA on CT and MRI scans using Scheltens’ MTA scale. Ratings were compared to a consensus rating by two experienced neuroradiologists for estimation of true positive and negative rates (TPR and TNR) and over- and underestimation of MTA. Inter-modality agreement expressed as Cohen’s κ (dichotomized data), Cohen’s κ_w_, and two-way mixed, single measures, consistency ICC (ordinal data) were determined. Adequate agreement was defined as κ/κ_w_ ≥ 0.80 and ICC ≥ 0.80 (significance level at 95% CI ≥ 0.65).

**Results:**

Forty-nine subjects (median age 72 years, 27% abnormal MTA) with cognitive impairment were included. Only junior radiologists achieved adequate agreement expressed as Cohen’s κ. All raters achieved adequate agreement expressed as Cohen’s κ_w_ and ICC. True positive rates varied from 69 to 100% and TNR varied from 85 to 100%. No under- or overestimation of MTA was observed. Ratings did not differ between radiologists.

**Conclusion:**

We conclude that radiologists with varying experience achieve adequate inter-modality agreement and similar accuracy when Scheltens’ MTA scale is used to rate MTA on a non-demented population. However, TPR varied between radiologists which could be attributed to rating style differences.

**Key Points:**

• *Radiologists with varying experience achieve adequate inter-modality agreement with similar accuracy when Scheltens’ MTA scale is used to rate MTA on a non-demented population*.

• *Differences in rating styles might affect accuracy, this was most evident for senior neuroradiologists, and only junior radiologists achieved adequate agreement on dichotomized (abnormal/normal) ratings*.

• *The use of an MTA scale template might compensate for varying clinical experience which could make it applicable for clinical use*.

## Introduction

Medial temporal lobe atrophy is an important structural finding associated with Alzheimer’s disease but MTA can be found in preclinical dementia [1–5]. In clinical practice, the interpretation of structural imaging is based on the visual assessment by radiologists. However, MTA is underreported even when assessment is warranted which might have clinical implications [6, 7]. Introducing structured reporting using visual rating scales (VRS) has been shown to increase reporting frequency of MTA, however with little effect on accuracy, and also affecting diagnostic outcome in the diagnostic workup of cognitive impairment [8, 9]. Scheltens’ scale for visual rating of MTA is also endorsed in the diagnostic workup of cognitive impairment [9–11].

A recent European survey showed that 75% of responding centers used VRS in clinical practice where white matter changes scales were regularly used by 82% and the MTA scale by 81%; other VRS were less common. Lack of training was the main reason for not using VRS in clinical practice [12]. It has been suggested that using VRS in practice might increase accuracy and yield more complete reports [9]. An alternative to visual rating would be quantitative volumetric measurements that although common in research were regularly used in only 6% of responding centers [12]. Although visual rating of MTA is more widespread, radiologists require regular training to maintain a high intra-rater agreement while inter-rater agreement can remain low [13]. Additionally, rating style differences could also result in low inter-rater agreement even across experienced radiologists which in turn could affect accuracy where raters consequently giving lower MTA grades could consequently underestimate abnormal MTA [5]. Inter-modality agreement for MTA has previously been assessed in demented populations with substantial to almost perfect agreement [14, 15].

However, ratings have not been compared to a gold standard rating of MTA for assessment of accuracy and inter-modality agreement has not yet been assessed in a non-demented population with mild cognitive symptoms (MCS). We hypothesized that the use of an MTA template might reduce the effect of rating style differences and yield adequate agreement across radiologists with varying clinical experience. Our aims were therefore (1) to assess inter-modality agreement for MTA ratings in a non-demented population in order to mimic the everyday clinical situation and (2) to assess accuracy across radiologists with varying clinical experience, given access to an MTA scale template during ratings.

## Materials and methods

This observational study was performed at a single center on a population recruited from the Swedish BioFINDER (Biomarkers for Identifying Neurodegenerative Disorders Early and Reliably) study MCS cohort (see https://biofinder.se). Eligible subjects were enrolled between the years 2010 and 2014, had a Mini-Mental State Examination score of 24 to 30, aged 60 to 80 years, and did not meet the criteria for dementia [16]. All eligible subjects had performed a baseline magnetic resonance imaging (MRI) as part of the BioFINDER study and a clinical routine computed tomography (CT) as part of a dementia diagnostic workup 100 days prior to this MRI. All CT exams were retrospectively recruited from our picture archiving and communication system (PACS) (IDS7®, Sectra AB).

**Fig. 1:**
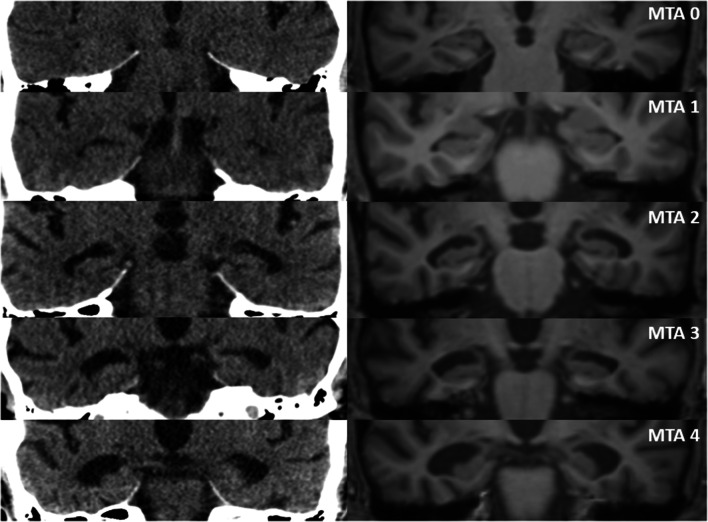
Image examples from MTA grades according to Scheltens’ ordinal 5-point MTA scale (CT to the left). Each side was rated separately, and the overall highest score was given. MTA 0 = no atrophy, MTA 1 = mild widening of choroid fissure, MTA 2 = moderate widening of choroid fissure *and* mild widening of temporal horns *and* mild decrease in hippocampal height, MTA 3 = severe widening of choroid fissure *and* moderate widening of temporal horns *and* moderate decrease in hippocampal height, and MTA 4 = severe widening of choroid fissure *and* severe widening of temporal horns *and* severe decrease in hippocampal height. Abbreviations: MTA, medial temporal lobe atrophy

Scheltens’ ordinal 5-point MTA scale was used to grade MTA accordingly: MTA 0 = no atrophy; MTA 1 = mild widening of choroid fissure; MTA 2 = moderate widening of choroid fissure *and* mild widening of temporal horns *and* mild decrease in hippocampal height; MTA 3 = severe widening of choroid fissure *and* moderate widening of temporal horns *and* moderate decrease in hippocampal height; MTA 4 = severe widening of choroid fissure *and* severe widening of temporal horns *and* severe decrease in hippocampal height [10, 17]. Image examples of MTA grades corresponding to Scheltens’ MTA scale are presented in Fig. 1.

Each side was rated separately, and the overall highest score was given. Results were dichotomized into abnormal (2–4 if < 75 years, 3–4 if ≥ 75 years) and normal (0–1 if < 75 years, 0–2 if ≥ 75 years), consistent with our clinical routine but also to allow for comparison with previous studies [6–8].

Four raters—one last year resident in radiology (“Rater 1”), one senior neuroradiologist with > 30 years’ experience (“Rater 2”), one radiologist on first year of a residency in neuroradiology (“Rater 3”), and one senior neuroradiologist with seven years’ experience (“Rater 4”)—performed ratings of all CT and MRI scans with 2 weeks apart. For group comparison, “Rater 1” and “Rater 3” are denoted “junior radiologists” and “Rater 2” and “Rater 4” are denoted “senior neuroradiologists.” All raters had access to an MTA template (published by Mårtensson et al in [18]) during ratings but were given no further training. Raters were blinded to all clinical information. Additionally, two experienced neuroradiologists (not raters in the study) performed a dichotomized (i.e., normal/abnormal), joint consensus assessment of all exams which served as our gold standard.

The MRI scans were performed on a 3-T (Trio®, Siemens AG) scanner where coronal T1-weighted three-dimensional magnetization prepared rapid acquisition with gradient echo, echo time 3 ms, repetition time 1950 ms, flip angle 9°, acquisition matrix 356 × 215, and pixel spacing 0.98/0.98 were obtained. The CT scans were performed on scanners from three different vendors with 120-kV voltage, 150 to 320-mAs exposure, 0.5 to 0.75 collimation, and 0.36 to 0.65 pitch factor. Only subjects where original 0.75-mm axial slices were available for reformatting in our PACS were eligible for inclusion. All scans were reformatted into oblique slices perpendicular to the hippocampi with 3-mm thickness and no gap. All images were assessed in our PACS where CT images were assessed with a center width of 40 HU and a window width of 80 HU.

### Statistics

For assessment of inter-modality agreement on dichotomized data, Cohen’s κ was estimated. For assessment of inter-modality agreement on ordinal data, Cohen’s weighted κ (κ_w_) with quadratic weighting was estimated. For assessment of average agreement on ordinal data for several (> 2) raters, two-way mixed, single measure, consistency intraclass correlation coefficients (ICC) were estimated as a substitute for Cohen’s κ_w_ [19]. To estimate over- and underestimation of MTA compared to our gold standard, the McNemar test for comparison of paired categorical data was used. For all tests, *p* < 0.05 was considered significant. In order to assess accuracy, true positive rates (TPR) and true negative rates (TNR) were estimated for each radiologist with 95% CIs using MEDCALC® (MedCalc Software Ltd.) online statistics calculator (https://www.medcalc.org/calc/diagnostic_test.php). Sample size was estimated in accordance with a confidence interval (CI) construction for κ-estimations suggested by Donner and Rotondi and performed using the “kappaSize” package in R [20, 21]. Adequate agreement was defined as κ or ICC ≥ 0.80 with the significance level set at 95% CI ≥ 0.65. For all other calculations, SPSS® (IBM Corp) version 26 was used.

## Results

The abovementioned criteria for sample size estimation resulted in the requirement of a sample size of at least 46 subjects. Our search identified 50 eligible subjects; one subject with enlarged ventricles secondary to reasons other than atrophy was excluded, and thus 49 subjects were included. The prevalence of abnormal MTA was 27% according to our gold standard (see further data in Table 1).

**Table 1:** Basic data on our study population

Characteristic	
Total (n)	49
Age in years (median, IQR)	72 (7.4)
Gender (female, %)	47
Days between CT and MRI (median, IQR)	79 (32)
Prevalence of abnormal MTA (%)*	27

*Note*: Data rounded to nearest integer where applicable. *Consensus rating by two experienced neuroradiologists (not raters in this study). *Abbreviations*: *MTA*, medial temporal lobe atrophy; *IQR*, interquartile range

**Fig. 2:**
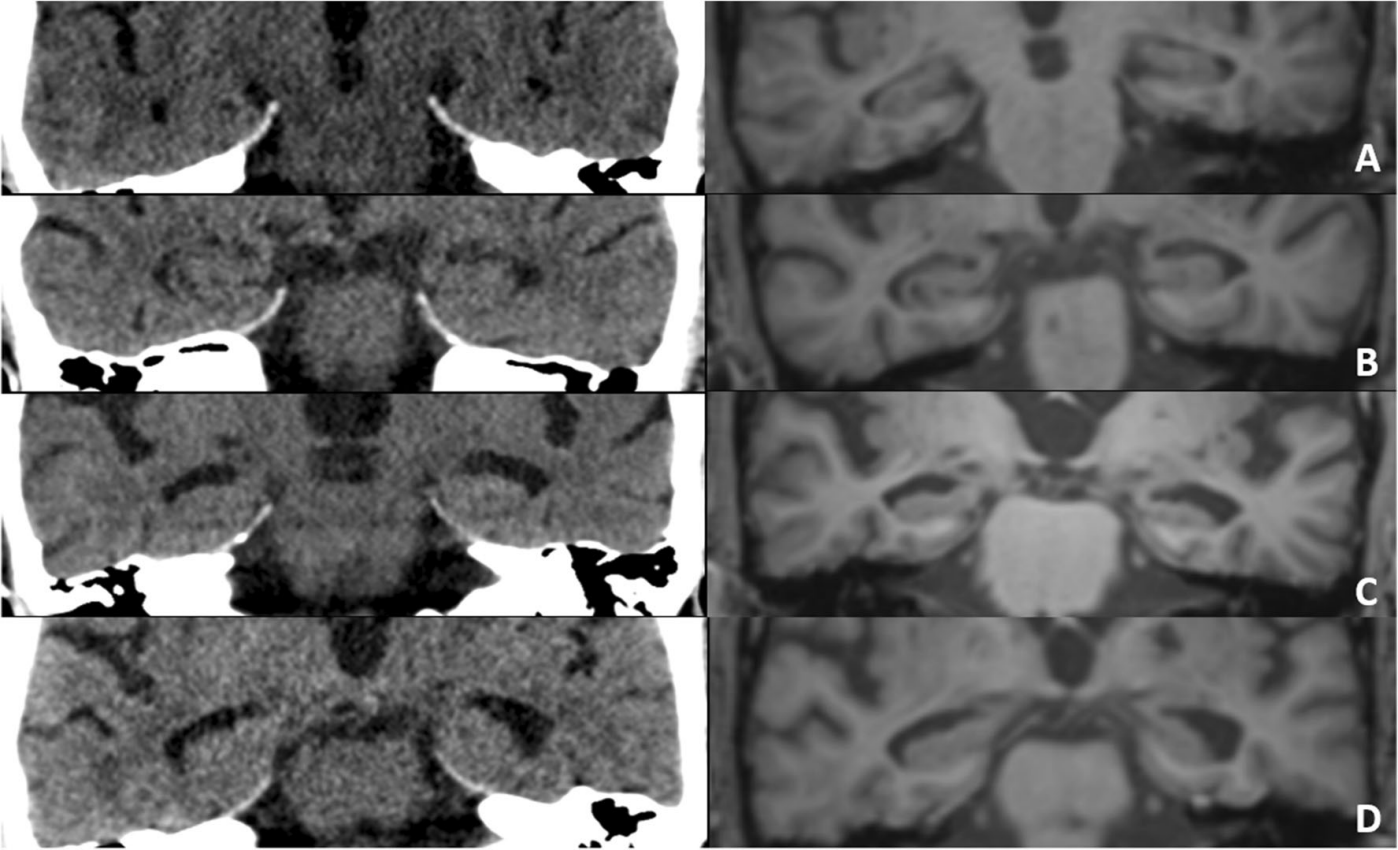
Image examples (CT to the left) of rater discrepancies for four subjects. Each side was rated separately, and the overall highest score was given. **Subject** “**A**”: MTA was graded “0” by two raters and graded “1” by two raters on both CT and MRI. **Subject** “**B**”: MTA was graded “1” by two raters and “2” by two raters on CT; MTA was graded “1” by one rater and “2” by three raters on MRI. **Subject** “**C**”: MTA was graded “2” by two raters and “3” by two raters on CT; MTA was graded “3” by all raters on MRI. **Subject** “**D**”: MTA was graded “2” by one rater and “3” by three raters on CT; MTA was graded “3” by three raters and “4” by one rater on MRI. Abbreviations: MTA, medial temporal lobe atrophy

Expressed as Cohen’s κ, inter-modality agreement was adequate (κ ≥ 0.80, 95% CI ≥ 0.65) for “Rater 1” and “Rater 3” (junior radiologists) but not for “Rater 2” and “Rater 4” (senior neuroradiologists). Inter-modality agreement on ordinal ratings expressed as Cohen’s κ_w_ was adequate (κ_w_ ≥ 0.80, 95% CI ≥ 0.65) for all raters (see Table 2). Average agreement expressed as ICC was adequate on MRI and CT ratings for all raters (ICC ≥ 0.80, 95% CI ≥ 0.65) (see Table 3). Image examples of rater discrepancies are shown in Fig. 2.

**Table 2:** Inter-modality agreement for each rater

	Rater 1	Rater 2	Rater 3	Rater 4
Cohen’s κ	**0.95**	0.80	**0.85**	0.75
Cohen’s κ_w_	**0.95**	**0.87**	**0.88**	**0.83**

*Note*: Dichotomized data for Cohen’s κ assessment and ordinal data for Cohen’s κ_w_* assessment. *Quadratic weight*.* **Bold** indicates adequate agreement (κ ≥ 0.80, 95% CI ≥ 0.65). *Abbreviations*: *CI*, confidence interval

**Table 3:** Average agreement on ordinal ratings expressed as intraclass correlation coefficients

	ICC*		
	MRI	CT	MRI and CT
All raters	**0.88**	**0.86**	**0.86**
Junior radiologists^1^	**0.92**	**0.87**	**0.89**
Senior neuroradiologists^2^	**0.86**	**0.84**	**0.85**

*Note*: *Two-way mixed, single measure, consistency ICC on ordinal data. ^1^“Rater 1” and “Rater 3”. ^2^“Rater 2” and “Rater 4”. **Bold** indicates adequate agreement (ICC ≥ 0.80, 95% CI ≥ 0.65). *Abbreviations*: *CI*, confidence interval; *ICC*, intraclass correlation coefficient

Compared to our gold standard, TPR varied from 77 to 100% on MRI and from 69 to 92% on CT. True negative rates varied from 94 to 100% on MRI and from 89 to 100% on CT. Junior radiologists performed somewhat better compared to senior neuroradiologists, but the differences were not significant based on visual comparison of 95% CIs (see Table 4). Overestimations of abnormal MTA were observed for “Rater 1,” “Rater 3,” and “Rater 4” on CT and for “Rater 1” and “Rater 3” on MRI. Underestimations of abnormal MTA were observed for “Rater 2” on CT and for “Rater 2” and “Rater 4” on MRI. None of these observations were significant compared to our gold standard (see Table 5).

**Table 4:** True positive rate and true negative rate for each rater (n = 49)

	Rater 1	Rater 2	Rater 3	Rater 4
	MRI			
TPR %, (95% CI)	92 (64 to 100)	77 (46 to 95)	100 (75 to 100)	85 (55 to 98)
TNR %, (95% CI)	94 (81 to 99)	100 (90 to 100)	94 (81 to 99)	97 (85 to 100)
	CT			
TPR %, (95% CI)	92 (64 to 100)	69 (39 to 91)	85 (55 to 98)	85 (55 to 98)
TNR %, (95% CI)	92 (78 to 98)	100 (90 to 100)	92 (78 to 98)	89 (74 to 97)

*Note*: Data rounded to nearest integer. There were no significant differences between raters based on visual comparison of 95% CIs. *Abbreviations*: *TPR*, true positive rate; *TNR*, true negative rate; *CI*, confidence interval

**Table 5:** Estimations of abnormal MTA for each rater compared to our gold standard (n = 49)

	Rater 1	Rater 2	Rater 3	Rater 4	Gold standard^1^
Abnormal MTA					
MRI	29%*	20%*	31%*	24%*	27%
CT	31%*	18%*	29%*	31%*	27%

*Note*: Data rounded to nearest integer. Percentages of assessed abnormal MTA for each rater compared to gold standard. *p > 0.05 (McNemar test) indicating no significant over- or underestimation compared to gold standard. ^1^Consensus rating of all exams by two experienced neuroradiologists (not raters in this study). *Abbreviations*: *MTA*, medial temporal lobe atrophy

## Discussion

In this observational study, we assessed inter-modality agreement and accuracy of MTA ratings across raters with varying clinical experience. Adequate agreement was achieved by junior radiologists on dichotomized ratings and by all raters on ordinal ratings. There was no significant over- or underestimation of MTA. Junior radiologists reached better accuracy compared to senior neuroradiologists, but the differences were not significant.

Few comparable studies on inter-modality agreement of MTA ratings are available. In the often-cited study by Wattjes et al, inter-modality agreement was assessed in 30 subjects where fourteen had clinical AD and eight had subjective memory impairment. Observed Cohen’s κ_w_ for three raters with varying experience ranged from 0.80 to 0.92 [14]. In a later study, Thiagarajan et al assessed inter-modality agreement in a subsample cohort of 107 subjects where 53 had known dementia and observed a Cohen’s κ_w_ of 0.74 [15]. None of these studies clearly specified if quadratic Cohen’s κ_w_ was performed, and an estimation of adequate sample size was not presented. Although our results are in line with these studies, further comparison should therefore be done with caution.

Previous studies have shown that MTA is severely underreported in clinical reports and introducing VRS in clinical practice has been shown to increase the reporting of MTA with an improvement of TPR from 10 to 55% in clinical reports [6–8]. A prospective study has also shown that the use of VRS in the assessment of structural imaging has an impact on the diagnostic outcome in suspected dementia why the use of VRS is warranted [9]. Our results showed an average TPR for four raters of at least 88% and no underestimation of MTA indicating that using a template might have positive effects on accuracy and agreement. This reasoning assumes that greater coherence and accuracy of radiology reports in turn might have an impact on the final diagnosis. The actual impact of increased reporting of MTA on the final clinical diagnosis was however not assessed within this study and further assessment of this issue is thus warranted.

Agreement does not consider if assessments are correct or not and should therefore be interpreted together with some assessment of accuracy [22]. We chose to assess TPR and TNR and over- and underestimation of MTA compared to our gold standard. Although some variations in TPR and TNR and some variations in over- and underestimation of MTA were observed, none were significant which might suggest that all raters were reliable in their assessments. We observed TPR from 85 to 100% and TNR from 89 to 97% for three raters (“Rater 1,” “Rater 3,” and “Rater 4”). By comparison, “Rater 2” (senior neuroradiologist) achieved a lower TPR of 69% on CT and 77% on MRI as well as a higher degree of underestimation of MTA which could be a result of “Rater 2” giving overall lower MTA grades. Additionally, “Rater 4” (senior neuroradiologist) did not achieve adequate agreement on dichotomized ratings which could imply a lesser rating consistency compared to the other radiologists. Thus, the senior neuroradiologists (“Rater 2” and “Rater 4”) seemed less consistent in their assessments compared to each other as well as compared to the junior radiologists which could be explained by rating style differences. It is possible that the junior radiologists had a stronger adherence to the MTA template while the senior neuroradiologists instead relied more on their clinical experience. A previous study by Mårtensson et al has shown that differences in rating styles result in low agreement between experienced radiologists even when they are strongly correlated compared to volumetry [5]. If a radiologist consequently gives lower or higher MTA scores, it could potentially result in under- or overreporting of MTA which could have a potential clinical impact. Reducing the clinical impact of rating style differences might thus affect agreement and accuracy of clinical reports. Since none of the differences in our study were significant, it could be argued that using a template might have reduced this effect, but since we did not assess how raters without access to a template would perform, any conclusions should be drawn with caution.

The κ-statistic is widely used as a measure of agreement in scientific radiology papers. Agreement using the κ-statistic is however highly susceptible to various biases, including prevalence, sample size, rater experience, and arbitrary interpretations which hampers comparisons between studies [19, 22–25]. McHugh has raised concern over acceptable κ-levels in health research and argued that data reliability must be considered. As an example, strong agreement, κ 0.80–0.90 according to McHugh, would correspond to correct agreement in 64 to 81% of cases and accepting lower κ-levels would result in accepting faulty or incoherent ratings in more than 36% of the cases [23]. We therefore believe that a minimum of strong agreement (i.e., κ ≥ 0.80) is warranted in clinical practice and defined adequate agreement accordingly. Rather than comparing to chance agreement as the null value, which is of limited value in a clinical context, we took reliability into account and chose a conservative approach with a significance level set at 95% CI ≥ 0.65 [23, 24].

Visual assessment of MTA is inherently subjective which hampers conclusions based on such observations as well as comparisons between studies. Quantitative measurements, i.e. volumetry or automated ratings, are often used in research settings but have not yet made their way into clinical practice [12]. Recent studies have shown reasonable to strong correlation for visual rating of MTA and quantitative measurements of hippocampal volume [5, 26–28]. Although visual ratings have been shown to be reliable compared to volumetry on follow-up of subjects with MCS, they are coarser and would require a longer follow-up time to detect a change in MTA by one grade [5, 28]. Additionally, correlation should not be used as a substitute for agreement since raters with strong correlation compared to volumetric measurements have been shown to have low levels of agreement [5].

This study has some limitations. (i) Sample size was considered sufficient for the κ-analysis; however, it is still relatively small which hampers the interpretation of TPR and TNR and over- and underestimation of MTA, respectively. (ii) We assessed a non-demented population with MCS which we believe better reflects the everyday clinical situation. This approach would raise concern if our intention had been to assess the diagnostic accuracy of the MTA scale, but since our aim was only to assess agreement, we considered our approach sufficient for this study. (iii) Visual ratings are subjective, and it would be preferable to use an automated rating as gold standard or at least compare human ratings to automated ratings for correlation and agreement. No automated ratings applicable on both CT and MRI and available for clinical use were known to us at the time of conceptualization and finalization of this study. We therefore chose a gold standard based on consensus ratings by two experienced neuroradiologists, but this approach could itself result in a potential bias. (iv) The retrospective recruitment of clinical CT exams resulted in scans being performed on different scanners. Although this is a reality in the everyday clinical situation, a potential bias from differences in image quality cannot be excluded. (v) We have not evaluated how raters would perform without access to a template or if proper training had preceded the ratings. However, knowledge of participation in a study where agreement is concerned could itself result in a hampering rater bias. Additionally, we have not assessed how raters at other institutions would perform under the same circumstances.

We hypothesized that using an MTA template could reduce the effect of rating style differences and yield adequate agreement across radiologists with varying clinical experience. Our results showed that adequate inter-modality agreement, with no significant differences across radiologists, was achieved when an MTA template was used. Using a template might thus compensate for varying experience and rating style differences. We believe our results further strengthen the already endorsed use of the MTA scale in clinical practice [6–9, 11].

In summary we conclude that radiologists with varying clinical experience achieve adequate inter-modality agreement with similar accuracy when Scheltens’ MTA scale is used to rate MTA on a non-demented population. However, TPR varied between raters which could be attributed to rating style differences.

## Abbreviations

BioFINDER: Biomarkers for Identifying Neurodegenerative Disorders Early and Reliably
CI: Confidence interval
CT: Computed tomography
ICC: Intraclass correlation coefficient
IQR: Interquartile range
MCS: Mild cognitive symptoms
MRI: Magnetic resonance imaging
MTA: Medial temporal lobe atrophy
PACS: Picture archiving and communication system
TNR: True negative rate
TPR: True positive rate

## Declarations

### Guarantor

The scientific guarantor of this publication is Danielle van Westen.

### Statistics and biometry

One of the authors has significant statistical expertise.

### Study subjects or cohorts overlap

Some study subjects or cohorts have been previously reported in Håkansson et al *Neurology* 2019; 61:397-404, and Håkansson et al *Eur Radiol* 2021; 31:666-673.

### Methodology

• Retrospective

• Observational

• Performed at one institution
